# Cognitive effects of Lewy body pathology in clinically unimpaired individuals

**DOI:** 10.1038/s41591-023-02450-0

**Published:** 2023-07-18

**Authors:** Sebastian Palmqvist, Marcello Rossi, Sara Hall, Corinne Quadalti, Niklas Mattsson-Carlgren, Sofia Dellavalle, Pontus Tideman, Joana B. Pereira, Maria H. Nilsson, Angela Mammana, Shorena Janelidze, Simone Baiardi, Erik Stomrud, Piero Parchi, Oskar Hansson

**Affiliations:** 1 Clinical Memory Research Unit, Department of Clinical Sciences, Malmö, Lund University, Lund, Sweden; 2 Memory Clinic, Skåne University Hospital, Malmö, Sweden; 3 IRCCS Istituto delle Scienze Neurologiche di Bologna, Bologna, Italy; 4 Department of Neurology, Skåne University Hospital, Lund University, Lund, Sweden; 5 Wallenberg Center for Molecular Medicine, Lund University, Lund, Sweden; 6 Department of Health Sciences, Lund University, Lund, Sweden; 7 Department of Biomedical and Neuromotor Sciences, University of Bologna, Bologna, Italy

**Keywords:** Neurodegeneration, Alzheimer's disease, Parkinson's disease

## Abstract

α-Synuclein aggregates constitute the pathology of Lewy body (LB) disease. Little is known about the effects of LB pathology in preclinical (presymptomatic) individuals, either as isolated pathology or coexisting with Alzheimer’s disease (AD) pathology (β-amyloid (Aβ) and tau). We examined the effects of LB pathology using a cerebrospinal fluid α-synuclein-seed amplification assay in 1,182 cognitively and neurologically unimpaired participants from the BioFINDER study: 8% were LB positive, 26% Aβ positive (13% of those were LB positive) and 16% tau positive. LB positivity occurred more often in the presence of Aβ positivity but not tau positivity. LB pathology had independently negative effects on cross-sectional and longitudinal global cognition and memory and on longitudinal attention/executive function. Tau had cognitive effects of a similar magnitude, but these were less pronounced for Aβ. Participants with both LB and AD (Aβ and tau) pathology exhibited faster cognitive decline than those with only LB or AD pathology. LB, but not AD, pathology was associated with reduced sense of smell. Only LB-positive participants progressed to clinical LB disease over 10 years. These results are important for individualized prognosis, recruitment and choice of outcome measures in preclinical LB disease trials, but also for the design of early AD trials because >10% of individuals with preclinical AD have coexisting LB pathology.

## Abstract (web summary)

A longitudinal study of clinically unimpaired individuals reveals that Lewy body pathology measured in vivo is associated with worse smell and cognitive functions and predicted subsequent cognitive decline and progression to Parkinson’s disease or dementia with Lewy bodies.

## Main

Lewy body pathology, the primary pathology of Lewy body disease (LBD), comprises the intraneuronal accumulation of aggregates of misfolded alpha-synuclein (α-syn) as LB and neurites^1,2^. The term LBD is used to describe mainly Parkinson’s disease (PD) and dementia with LB (DLB)^1,2^. Importantly, LB pathology is also often found as a copathology in AD^3^. According to studies using postmortem immunohistochemistry, LB pathology is present in approximately 8–12% of neurologically and cognitively unimpaired elderly individuals (‘incidental LBD’) with mean ages between 70 and 75 years^4–6^. Because of the previous lack of accurate biomarkers for misfolded α-syn, little is known about the effects of LB pathology in this preclinical (also known as ‘presymptomatic’) phase of LBD. However, recent advances have shown that misfolded α-syn can be detected in the cerebrospinal fluid (CSF) of patients with LB pathology using in vitro seed amplification assay (SAA) of α-syn^7^. This novel method has shown very high sensitivity and specificity in the detection of neuropathologically verified LBD, especially in individuals with limbic and cortical LBs^8–10^ and for clinically diagnosed PD^11^. There are no studies using this method to examine the effects of LB pathology on cross-sectional and longitudinal changes in clinical symptoms in neurologically and cognitively unimpaired individuals, neither as an isolated pathology nor coexisting with AD pathology. Knowing these effects is of great importance in the design of preclinical LBD trials, specifically to plan the screening procedure and to choose sensitive outcome measures. The coexistence of LB and AD pathologies is also important considering the many ongoing preclinical AD trials enrolling cognitively unimpaired participants with AD pathology. If LB pathology has cognitive effects similar to AD pathology, trials that account for the presence of LB pathology could better detect the effects of anti-Aβ treatments. As a copathology to AD, accounting for the presence of LB pathology may also be relevant when examining the cognitive trajectories of preclinical individuals in observational studies according to the AD framework that characterizes the presence of Aβ (A), tau (T) and non-AD specific neurodegeneration (N) for diagnosis^12^ and prognosis^13^.

In this study we aimed to examine the earliest effects of LB pathology on cognitive and other clinical outcomes and compare them to the effects of Aβ and tau pathologies. The cognitive outcomes were examined cross-sectionally and longitudinally up to 10 years in 1,182 cognitively and neurologically unimpaired participants from the BioFINDER-1 (NCT01208675) and BioFINDER-2 (NCT03174938) studies. In addition, a survival analysis was performed examining subsequent progression to DLB or PD based on the presence of LB pathology at baseline.

## Results

### Participants and prevalence of AD and LB pathologies

**Fig. 1:**
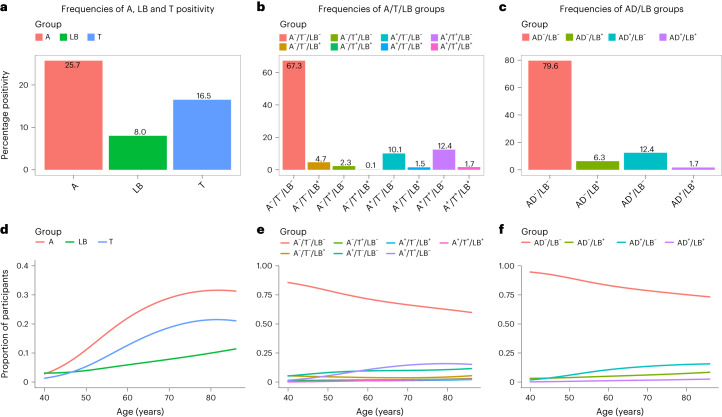
Prevalence of Aβ, tau and LB pathologies. **a**, Prevalence of Aβ (A), tau (T) and LB positivity. **b**, Prevalence of A/T/LB groups. **c**, Prevalence of AD/LB groups. **d**–**f**, Proportions of groups **a**–**c**, respectively, with increasing age, where **d** shows A, LB and T positivity, **e** combinations of A/T/LB positivity/negativity and **f** combinations of AD/LB positivity/negativity. Note that AD positivity refers to being both A^+^ and T^+^ while LB positivity refers to being α-syn SAA^+^. **d**, Using age as independent variable and pathology as dependent in logistic regression models, age had an OR of 1.066 (95% CI 1.036–1.099) for LB, 1.067 (95% CI 1.048–1.087) for A and 1.071 (95% CI 1.048–1.095) for T pathology.

Participant characteristics are shown in Table 1. AD pathology was defined as being Aβ and tau positive using CSF or positron emission tomography (PET) methods according to the National Institute of Aging–Alzheimer’s Association criteria^12^. LB pathology was defined as being α-syn SAA^+^ using a real-time, quaking-induced conversion assay (RT-QuIC)^8^. Note that the α-syn SAA we applied detects only the LB pathology seen in PD and DLB but not the presence of α-syn in the rare α-syn disorder multiple systemic atrophy^10^. Biomarker status was determined at study baseline. The prevalence of Aβ, tau and LB pathologies is shown in Fig. 1a–c. We found that 94 (8%) were LB^+^, 304 (26%) Aβ^+^ and 195 (16%) tau^+^, and 38 were both Aβ^+^ and LB^+^ (13% of Aβ positives). When defining AD pathology as being both Aβ^+^ and tau^+^ (ref. ^12^), we found that 941 (80%) participants had no AD nor LB pathology (AD^–^/LB^–^), 74 (6%) only LB pathology (AD^–^/LB^+^), 147 (12%) only AD pathology (AD^+^/LB^–^) and 20 (2%) both AD and LB pathology (AD^+^/LB^+^). All pathologies were more common with increasing age (see Fig. 1d–f and odds ratio (OR) in the figure legend). Adjusted for age, males were more than two times more likely to be LB^+^ compared with females (OR 2.56, 95% confidence interval (CI) 1.66–3.99). No significant association between sex and Aβ or tau was found.

**Table 1:** Characteristics of AD/LB groups

Variable	AD^–^/LB^–^ (*n* = 941)	AD^–^/LB^+^ (*n* = 74)	AD^+^/LB^–^ (*n* = 147)	AD+/LB+ (*n* = 20)	Total (*n* = 1,182)
Age, years	69 (9.1)	73 (7.4)	73 (5.7)	75 (4.4)	70 (8.8)
Education, years	12 (3.4)	12 (3.2)	12 (3.6)	13 (4.7)	12 (3.5)
Sex, *n* female	572 (60.8%)	30 (40.5%)	89 (60.5%)	7 (35.0%)	698 (59.1%)
MMSE, points	29 (1.1)	29 (1.3)	29 (1.2)	28 (1.6)	29 (1.1)
Global cognition (*z*-score)	0.056 (0.75)	−0.470 (0.80)	−0.630 (0.83)	−0.940 (0.79)	−0.070 (0.81)
Memory (*z*-score)	−0.014 (0.99)	−0.560 (1.2)	−0.630 (1.3)	−1.000 (0.87)	−0.140 (1.1)
Attention/executive (*z*-score)	−0.056 (0.91)	−0.390 (0.86)	−0.440 (0.86)	−0.600 (0.96)	−0.130 (0.91)
Smell function (*z*-score)	0.000 (0.96)	−1.300 (1.3)	−0.045 (0.77)	−0.990 (0.53)	−0.090 (1.0)
Motor function (CIMP-QUEST, *z*-score)	0.010 (0.91)	−0.650 (1.4)	−0.380 (1.2)	−0.054 (0.60)	−0.082 (1.0)
Motor function (UPDRS-III, *z*-score)	0.066 (0.97)	−0.110 (1.2)	−0.220 (1.0)	−0.730 (1.0)	−0 (1.0)
Signs of REM sleep disorder, *n*^a^	24 (2.6%)^a^	7 (9.5%)^a^	2 (1.4%)^a^	0 (0%)^a^	33 (2.8%)^a^
A/T/LB, *n*					
A^–^/T^–^/LB^–^	795 (84.5%)	0 (0%)	0 (0%)	0 (0%)	795 (67.3%)
A^–^/T^+^/LB^–^	27 (2.9%)	0 (0%)	0 (0%)	0 (0%)	27 (2.3%)
A^+^/T^–^/LB^–^	119 (12.6%)	0 (0%)	0 (0%)	0 (0%)	119 (10.1%)
A^–^/T^–^/LB^+^	0 (0%)	55 (74.3%)	0 (0%)	0 (0%)	55 (4.7%)
A^–^/T^+^/LB^+^	0 (0%)	1 (1.4%)	0 (0%)	0 (0%)	1 (0.1%)
A^+^/T^–^/LB^+^	0 (0%)	18 (24.3%)	0 (0%)	0 (0%)	18 (1.5%)
A^+^/T^+^/LB^–^	0 (0%)	0 (0%)	147 (100%)	0 (0%)	147 (12.4%)
A^+^/T^+^/LB^+^	0 (0%)	0 (0%)	0 (0%)	20 (100%)	20 (1.7%)
CSF Aβ42/Aβ40	0.095 (0.022)	0.087 (0.024)	0.043 (0.0097)	0.039 (0.0081)	0.087 (0.028)
Aβ-PET (SUVR)^b^	0.50 (0.093)	0.54 (0.11)	0.81 (0.11)	0.83 (0.061)	0.52 (0.12)
CSF P-tau217 (pg ml^–1^)	5.9 (2.7)	7.1 (3.9)	26 (16)	43 (35)	10 (12)
Tau-PET (SUVR)^c^	1.1 (0.092)	1.1 (0.097)	1.6 (0.400)	2.1 (0.630)	1.2 (0.190)
CSF α-syn SAA positivity (LB pathology), *n*	0 (0%)	74 (100%)	0 (0%)	20 (100%)	94 (8.0%)

Data are shown as mean (s.d.) unless otherwise specified. Note that tau-PET and Aβ-PET data are from BioFINDER-2 only, and CSF P-tau217 and CSF Aβ42/Aβ40 are from BioFINDER-1.

^a^Based on whether the participant had been told that he/she seems to ‘act out his/her dreams’^48^. Data available only for BioFINDER-2.

^b^Measured in a composite neocortical ROI using [^18^F]flutemetamol with pons as reference region.

^c^Measured in a temporal meta-ROI using [^18^F]RO948 with inferior cerebellar cortex as reference region^46^.

### Cross-sectional associations with clinical outcomes

**Fig. 2:**
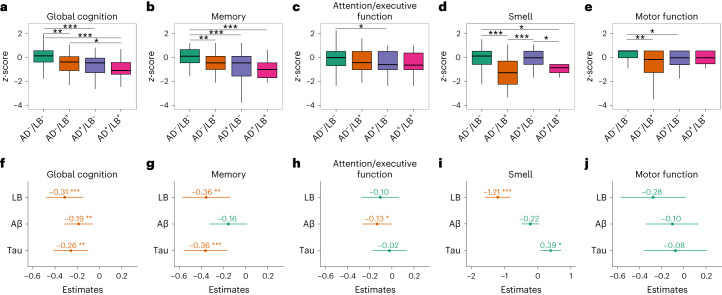
Comparisons between AD/LB groups and independent effects of LB, Aβ and tau pathologies on cross-sectional clinical outcomes. **a**–**j**, Significant effects (two-sided) were examined with linear regression models using either two AD/LB groups (**a**–**e**) or all three pathologies binarized (**f**–**j**) in the same model (to examine independent effects) while adjusting for age, sex and education (motor function was not adjusted for education). **a**,**f**, Global cognition. **b**,**g**, Memory. **c**,**h**, Attention/executive function. **d**,**i**, Smell. **e**,**j**, Motor function. Outcomes were *z*-scored cognitive tests (**a**–**c**,**f**–**h**), smell identification test (**d**,**i**) and an informant-based motor questionnaire (**e**,**j**). **a**–**e**, Boxes show interquartile range, horizontal lines are medians and whiskers were plotted using the Tukey method. **f**–**j**, Dot/center denotes estimate of the pathology and error bars 95% CI. Red indicates significant association between pathology and worse performance. In total, 941 participants were AD^–^/LB^–^, 74 AD^–^/LB^+^, 147 AD^+^/LB^–^ and 20 AD^+^/LB^+^; 94 were LB^+^, 304 Aβ^+^ and 195 tau^+^. Extended Data Fig. 2a,b shows the effect on motor function using the UPDRS-III scale (no significant effect of LB pathology). Statistical analyses with corrections for multiple comparisons are shown in Supplementary Fig. 1 (all effects of LB pathology were significant following correction). The effect of LB on clinical outcomes with/without adjustment for Aβ and tau is shown in Extended Data Table 1. Missing data shown in Supplementary Table 2. **h**, When restricting the analysis of attention/executive function to participants with available SMDT data (*n* = 854) the results were consistent, showing a significant effect for Aβ (*P* = 0.01) but not for tau and LB. *P < 0.05, **P < 0.01, ***P < 0.001 (two-sided).

**Extended Data Fig. 1:**
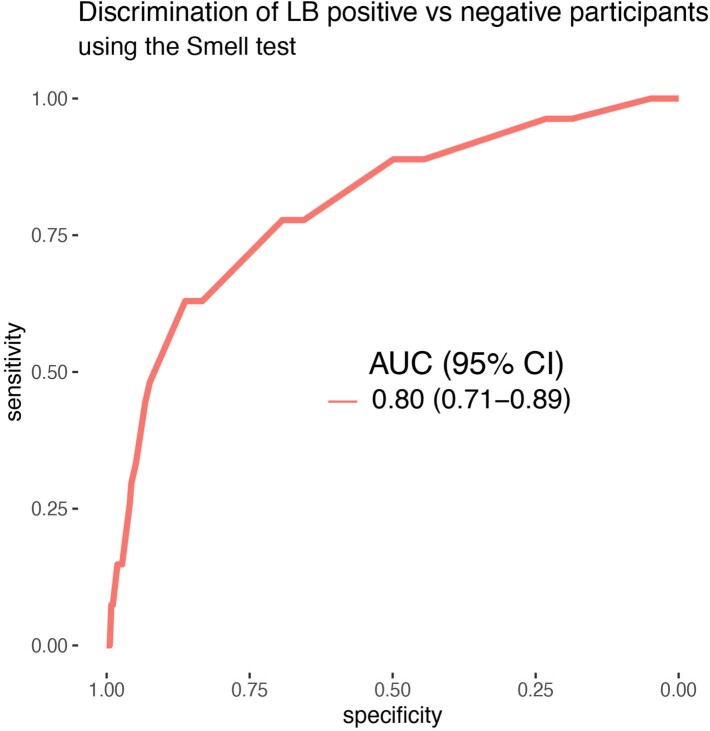
ROC analysis for discriminating LB positive versus LB negative participants using the Smell test. At the highest Youden index (−0.9 z-score on the smell test), the sensitivity was 63% and the specificity 86% with an overall percent correctly classified of 85%. Of those with available Smell test score (n = 398), 371 were LB- and 27 LB+.

When comparing cross-sectional group differences at baseline (Fig. 2a–e), participants that were AD^–^/LB^+^ or AD^+^/LB^–^performed significantly worse in baseline global cognition (measured with a preclinical Alzheimer cognitive composite (PACC)) and memory (measured with a ten-word delayed recall test) compared with those who were AD^–^/LB^–^. Moreover, AD^+^/LB^+^ participants had significantly worse global cognition compared with AD^–^/LB^+^. When examining the three pathologies as independent predictors in the same model, LB and tau pathologies had similar significant effects on lower global cognition and memory performance (Fig. 2f–h). On the other hand, Aβ pathology was the only pathology independently associated with worse attention/executive function but had no independent effect on memory function (Fig. 2g–h). The effect on memory was further examined in a recognition task, where only tau was significantly associated with worse performance (Supplementary Table 1). Reduced sense of smell was seen in participants that were AD^–^/LB^+^ or AD^+^/LB^+^ (Fig. 2d) and was specific to LB pathology (Fig. 2i). Its accuracy for LB pathology was further examined in receiving operating characteristic (ROC) analysis using LB status as outcome. The smell test predicted LB pathology with an area under the curve (AUC) of 0.80 (95% CI 0.71–0.89, overall accuracy, 85%; Extended Data Fig. 1).

**Extended Data Fig. 2:**
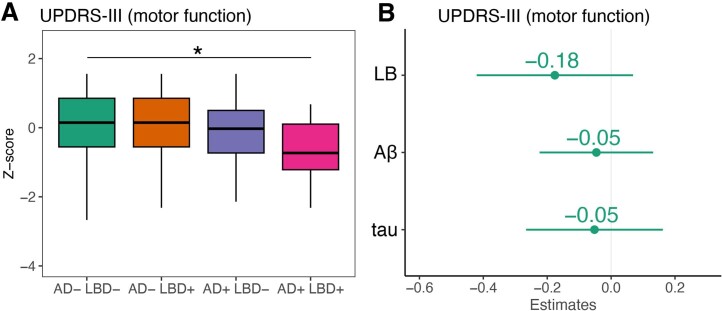
Comparison between AD/LB groups and independent effects of LB, Aβ and tau on motor function (UPDRS-III). The analyses were performed using linear regression models using two AD/LB groups (A) or all three pathologies binarized (B) in the same model (to examine independent effects), adjusted for age, sex, and UPDRS rater (three raters in total). The outcome was z-scored UPDRS-III values. Boxes in **a** show interquartile range, the horizontal lines are medians and the whiskers were plotted using the Tukey method. **b** shows the estimate of the pathology with 95% confidence interval. The statistical analyses with corrections for multiple comparisons are shown in Supplementary Fig. 2. 660 participants had UPDRS data, of whom 510 AD-/LB-, 48 AD-/LB+, 90 AD+/LB-, and 12 AD+/LB+. 60 were LB+, 209 Aβ+, and 122 tau+. Since UPDRS-III was not available for all participants, we performed a power analysis to examine what effect size the study was powered to detect (that is, effect of LB pathology on UPDRS in panel B). Using a power calculation for linear regression models, the smallest detectable effect size (f2) was 0.012, at 80% power and alpha=0.05. This suggests that our study was powered to detect even a small effect size of LB pathology on motor function. After applying multiple comparison correction with the FDR method (at α = 0.05), the AD+/LB+ group did not perform significantly different compared with AD-/LB- (Supplementary Fig. 2). * p < 0.05 (two-sided).

Worse motor function, measured using an informant-based questionnaire, was seen in those participants that were AD^–^/LB^+^ or AD^+^/LB^–^ compared with those that were AD^–^/LB^–^ but, when examining the independent effects of LB, Aβ and tau (in the same model) on motor function, none of the pathologies was significant at this preclinical disease stage (Fig. 2e,j). The absence of effect on motor function was also confirmed when using the unified Parkinson’s disease rating scale part III (UPDRS-III) as a measure of motor function (Extended Data Fig. 2).

The cross-sectional effect sizes of LB pathology on cognitive, smell and motor functions were similar regardless of whether the models were adjusted for Aβ and/or tau or not (Extended Data Table 1).

### Cross-sectional associations with pathologies and atrophy

There was a significant association between LB and Aβ status in that LB positivity was more likely to occur in the presence of Aβ positivity (*P* = 0.001 from the *χ*^2^ test), and also when adjusting for age (OR for LB, 1.72; *P* = 0.017). There was no significant association between LB and tau status (*P* = 0.15, *P* = 0.41 adjusted for age). Finally, LB status was not associated with magnetic resonance imaging measures of gray matter integrity in this preclinical population (see Methods for analyses).

### Longitudinal associations with cognitive outcomes

**Fig. 3:**
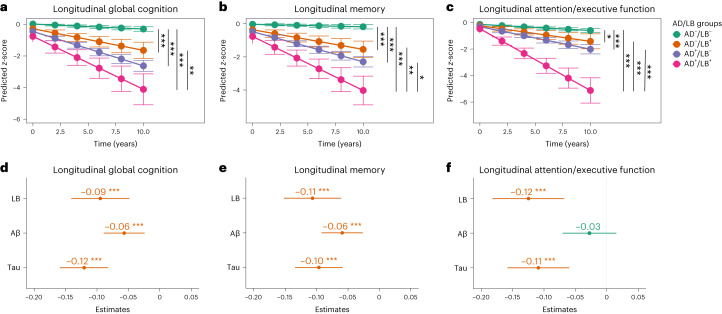
Independent effect of AD/LB groups and LB, Aβ and tau pathologies on longitudinal cognitive performance. **a**–**c**, Significant effects (two-sided) were examined with LME models focusing on the interaction of AD/LB group × time, adjusted for age, sex and education. **a**,**d**, Longitudinal global cognition. **b**,**e**, Longitudinal memory. **c**,**f**, Longitudinal attention/executive function. **d**–**f**, Interaction time × all three pathologies (binarized) was used in the same model to examine the independent effects of each pathology on cognitive progression while adjusting for age, sex and education. Outcomes were *z*-scored cognitive tests. **d**–**f**, Red indicates significant association between pathology and worse cognitive decline. The effect of LB on clinical outcomes with/without adjusting for Aβ and tau is shown in Extended Data Table 3. **a**–**c**, Estimated marginal means and 95% CI of means obtained from LME models by AD/LB group. **d**–**f**, Dot/center indicates the interaction estimate of time × pathology; error bars 95% CI. In total, 941 participants were AD^–^/LB^–^, 74 AD^–^/LB^+^, 147 AD^+^/LB^–^ and 20 AD^+^/LB^+^; 94 were LB^+^, 304 Aβ^+^ and 195 tau^+^. Statistical analyses with corrections for multiple comparisons are shown in Supplementary Fig. 3 (all significant differences/associations were still significant after correction). Missing data shown in Supplementary Table 2. *P < 0.05, **P < 0.01, ***P < 0.001 (two-sided).

The effect of the different AD/LB groups and the independent effects of all three pathologies (that is, LB, Aβ and tau) on longitudinal cognitive function were examined in linear mixed-effects (LME) models focusing on the interaction of time × AD/LB group (Fig. 3a–c) and time × pathology (absent/present; Fig. 3d–f). Group comparisons showed that those participants that were AD^–^/LB^+^, AD^+^/LB^–^ or AD^+^/LB^+^ progressed faster in all cognitive measures than those who were AD^–^/LB^–^ (Fig. 3a–c). Further, participants who were positive for both AD and LB pathologies (AD^+^/LB^+^) progressed significantly faster in memory and attention/executive function than those with only AD or LB pathology (AD^+^/LB^–^ or AD^–^/LB^+^) (Fig. 3b–c), and in global cognition compared with those with AD^–^/LB^+^ (Fig. 3a). To demonstrate that differences in trajectories were not caused by baseline group differences, we performed a sensitivity analysis using change in cognition as outcome and adjusted the model for baseline cognitive test score (Extended Data Table 2). This confirmed the significant differences shown in Fig. 3a–c. When studying the independent effects of LB, Aβ and tau pathologies on cognitive progression we found similar effects of LB and tau pathologies on all cognitive outcomes, but these were less pronounced for Aβ (Fig. 3d–f). The longitudinal LME models with/without adjusting for Aβ and tau are shown in Extended Data Table 3 (showing that the effect sizes of LB pathology were similar regardless of whether adjusting for AD pathology or not).

### LB pathology and progression to PD or DLB

**Fig. 4:**
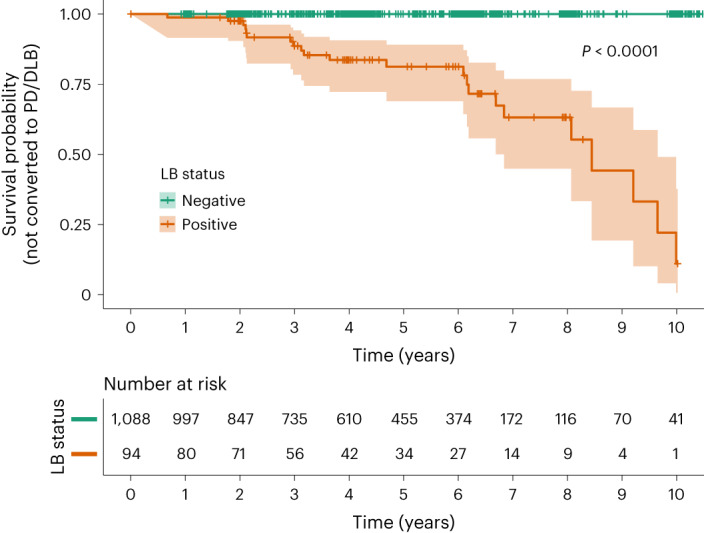
Survival curves for progression to PD or DLB stratified by LB status at baseline. The event was met when a participant fulfilled the clinical criteria for PD^49^ or DLB^50^ (alternatively, prodromal DLB^42^). Lines show point estimates of survival curves and shaded areas 95% CI. Vertical lines indicate time points of censoring. The table below shows the number of participants at each time point that had not yet progressed to PD/DLB. No participants who were LB^–^ at baseline progressed to PD/DLB. See Extended Data Table 4 for specifications of progression to a clinical diagnosis based on AD/LB positivity. *P* value derived from the log-rank test and indicates that the survival curves (that is, time to PD/DLB) of LB^+^ and LB^–^ participants are significantly different.

During a mean (s.d.) follow-up time of 4.46 (2.68) years of all study participants, 70 progressed to AD, 31 to vascular disease/dementia, 16 to DLB and six to PD irrespective of biomarker status at baseline (Extended Data Table 4). The survival analysis of progression to a clinical diagnosis of DLB or PD stratified on LB status at baseline is shown in Fig. 4. Only LB^+^ participants subsequently progressed to DLB or PD. The mean follow-up time of LB^+^ participants (*n* = 94) to the last visit or progression to DLB/PD was 3.92 (2.61) years, during which 23.4% progressed to either DLB or PD. Of those LB^+^ participants who did not progress to DLB/PD (*n* = 72), 14 (19.4%) developed LB pathology-related signs such as orthostatic hypotension (*n* = 9), signs of rapid eye movement (REM) sleep behavior disorder (*n* = 3) or signs of parkinsonism (*n* = 2) without fulfilling the clinical criteria for DLB or PD. In comparison, the smell test was not associated with a clinical follow-up diagnosis of DLB or PD, either as a binary predictor using survival analysis (*P* = 0.69) or as a continuous predictor in ROC analysis (AUC 0.64, 95% CI 0.45–0.83).

## Discussion

This study examines and compares the independent clinical effects of LB, Aβ and tau pathologies in a large population of cognitively and neurologically unimpaired individuals. Overall we found that LB pathology, detected by α-syn SAA in CSF, was present in 8% of the population (Fig. 1). It had similar effects on cross-sectional (Fig. 2) and longitudinal (Fig. 3) cognitive outcomes as AD pathology. Reduced smell function was specifically associated with LB pathology and a smell test could predict LB status with an AUC of 0.80 (Extended Data Fig. 1). Finally, survival analysis showed that only participants who were LB^+^ at baseline progressed to a clinical diagnosis of DLB or PD (Fig. 4).

The effect of LB pathology on memory function had a similar magnitude to that of tau pathology, both cross-sectionally (Fig. 2g) and longitudinally (Fig. 3e). As shown previously, the effect of tau on memory function in cognitively unimpaired individuals is related to early tau accumulation in the medial temporal lobe^14–16^. For LB pathology it could be related more to retrieval of memory than encoding, as indicated in our supplementary analysis showing that tau but not LB pathology was related to worse memory recognition (Supplementary Table 1). Although this result is preliminary due to the lower sample size, it is supported by previous results^17^. Other potential mechanisms could be the early involvement of LB pathology in the medial temporal regions^18^ or that LB pathology has a negative effect on dopamine^19^, which is important for memory encoding^20^.

The modest, isolated, cross-sectional effect of Aβ on attention/executive function agrees very well with recent results in cognitively unimpaired individuals^15^. This effect may reflect the early Aβ accumulation in regions important for attention and executive function that belong to the default mode and central executive networks^21^. The effect of LB pathology on global cognition measured with PACC (Figs. 2f and 3d), which is a measure used as a primary outcome in several preclinical AD trials^22^, is important to note. Not testing for LB positivity when including participants in such AD trials could hamper the ability to detect effects of anti-Aβ or anti-tau treatments on cognitive decline and could potentially be one of several causes for continuous cognitive decline despite complete removal of Aβ from the brain in AD participants^23,24^. Overall, we found that similar cognitive measures that are sensitive to changes in preclinical AD are also sensitive to LB pathology (Fig. 3) and could be suitable for preclinical LBD trials.

In the present study we found that 13% of Aβ^+^ participants were also LB^+^ (Table 1), and that LB positivity was significantly more likely to occur in the presence of Aβ positivity (OR 1.72) but not tau positivity. This is in agreement with neuropathology-based studies showing a clear overrepresented co-occurrence of AD and LB pathology in sporadic AD^25,26^.

Potential mechanisms for this co-occurrence could be a previously suggested seeding effect of Aβ on α-syn^27^ and indications that the *APOE* ε4 allele not only drives AD pathology but has also been associated with more severe LB pathology in AD^28,29^. Further, in autosomal dominant AD and Down syndrome, both of which have an overproduction of Aβ, LB pathology occurs more frequently than merely by chance (LB pathology prevalence of 50% in individuals with Down syndrome^30^ and 60% in autosomal dominant AD^31^ defined as LB pathology presence in at least the amygdala). This co-occurrence could be an issue when interpreting the effects of anti-AD treatments in Aβ^+^ cognitively unimpaired individuals, especially if LB^+^ participants are unequally distributed between treatment and placebo arms.

Regarding recruitment to future trials of preclinical LBD, detection of LB positivity will be crucial in identifying a relevant study population (for example for anti-LB therapy^32^). Our study shows that there is a relatively low prevalence of LB positivity (8%) in healthy middle-aged and elderly individuals, similar to that shown in recent smaller studies using α-syn SAA (3–10%) (refs. ^33,34^).

At the same time we found that progression to a clinical diagnosis of DLB or PD occurred only among participants who were LB^+^ at baseline, supporting the use of an α-syn SAA to identify preclinical LBD for early disease-modifying therapy trials in analogy to how Aβ and tau biomarkers are used today for preclinical AD trials. However, the low prevalence of LB pathology in unimpaired populations suggests that large screenings are needed to recruit adequately large populations, making CSF analyses a feasible, but not optimal, tool. The screening process would instead benefit from the use of blood or skin biopsies for α-syn SAA testing^35–37^, if they have comparable performance to CSF methods. Related to such a screening procedure, we found that the smell test of the present study had an AUC of 0.80 for discriminating LB^+^ versus LB^–^ participants (Extended Data Fig. 1). Although this is not sufficiently accurate for identification of LB pathology on its own, it indicates that smell tests, potentially combined with other measures, might be used in a prescreening procedure to identify suitable individuals for more expensive and invasive α-syn SAA testing. Our finding of an association between a smell test and LB pathology is supported by neuropathological studies showing that incidental (preclinical) LBD is associated with impaired sense of smell^38^. This is also found for DLB/PD dementia cases as well as AD with, but not without, significant LB pathology^39^. Over 50 studies have found that impaired sense of smell is a common feature in clinically diagnosed AD^40^, but our results suggest that this symptom is entirely caused by concomitant LB pathology (Fig. 2i).

Regarding motor function, no convincing effect of LB pathology was observed using either an informant-based questionnaire (Fig. 1j) or a motor function scale (UPDRS-III; Extended Data Fig. 2b). Although LB pathology has been linked to motor function in symptomatic individuals^41^, motor function may be affected at later stages in some cases with LBD as shown in prodromal LBD (that is, DLB at the mild cognitive impairment (MCI) stage and isolated REM sleep behavior disorder), where only about one-half of these patients have reduced dopamine transporter uptake in basal ganglia as demonstrated by single-photon emission computed tomography or PET^42^. Moreover, neither the informant-based motor questionnaire nor the UPDRS-III score may be sufficiently sensitive to capture the earliest signs of motor dysfunction. Future studies may benefit from the inclusion of digital assessments (for example, wearable sensors) and utilization of a dual-task paradigm for detection of subtle motor impairments.

Aβ and tau positivity have previously been shown to be strongly linked to near-term cognitive progression in cognitively unimpaired individuals^13^. The present study shows that detection of LB pathology in clinically unimpaired individuals also has prognostic implications, both in terms of progression to clinical DLB or PD (Fig. 4) and cognitive decline (Fig. 3). Cognitive decline is also supported by retrospective analyses based on neuropathological findings, highlighting for example the rapid progression in attention/executive function of AD^+^/LB^+^ individuals versus AD or LB pathology only^43,44^, similar to the present study where we show the additive effect of having both pathologies (Fig. 3a–c). Overall, this indicates that it could be equally important to measure the presence of LB pathology as measuring Aβ and tau, perhaps in an A/T/LB framework. Using this updated biomarker framework could further increase the understanding of the heterogeneity in clinical progression observed in participants classified according to the traditional framework using Aβ and tau pathologies only^45^.

This study has some limitations. Although the population was large (*n* = 1,182), few individuals were AD^+^/LB^+^ (*n* = 20), resulting in low statistical power to detect group differences compared with this group. Therefore, a lack of statistically significant difference versus this group should be interpreted with caution. This does not, however, affect the results of the independent effects of the three pathologies (*n* = 94 LB^+^, *n* = 304 Aβ^+^ and *n* = 195 tau^+^; Table 1), which overall confirmed the group analyses (Figs. 2f–j and 3d–f). Further, this study had missing data. That is, analyses were performed on all eligible participants and not on a restricted sample with complete data for all cognitive and noncognitive measures. The rationale for this was to not introduce a selection bias, but this resulted in missing data (detailed in Supplementary Tables 2 and 3). According to the study design, assessments of motor and smell function were included in only parts of the BioFINDER study. Despite this, significant associations were found between LB pathology and smell function (Fig. 2 and Extended Data Fig. 1). To examine whether the lack of an association with motor function could be due to lower statistical power (type II error) we performed a power analysis showing that, with the current sample (*n* = 660 with UPDRS data), we were powered to detect even a small effect size (Extended Data Fig. 2). Regarding the associations with cognition, it should be noted that the underlying cognitive tests used to measure these cognitive domains do not capture all aspects of that domain. Significant associations are thus dependent on the type of underlying tests used to measure a specific cognitive domain. Due to the study designs, baseline Aβ- and tau-PET were available only in BioFINDER-2 and thus CSF P-tau217 and CSF Aβ42/40 were instead used in BioFINDER-1 as biomarkers of Aβ and tau. These CSF biomarkers have, however, shown very high agreement with their PET counterparts^46,47^.

In summary, this study shows that LB pathology measured using α-syn SAA is associated with early changes in cognitive performance and smell function in cognitively and neurologically unimpaired individuals, and indicates an increased risk of near-term progression to DLB or PD. Further, 13% of clinically unimpaired Aβ^+^ individuals are also LB^+^ and the coexistence of both LB and AD pathologies has additional detrimental effects on cognition compared with either pathology alone. Therefore, these findings may have implications for the design of preclinical AD drug trials, where one could consider excluding LB^+^ individuals or ascertaining that the distribution of LB positivity is equal between treatment and placebo arms. Finally, the results can inform about the design of future preclinical LBD trials evaluating drugs targeting, for example, misfolded α-syn.

## Methods

### Participants

All participants were part of either the BioFINDER-1 (NCT01208675; *n* = 754) or BioFINDER-2 (NCT03174938; *n* = 428) study described previously^46,51,52^. Only participants with a complete dataset of Aβ, tau and α-syn data and who were cognitively and neurologically unimpaired at baseline were included. The classification of cognitively unimpaired was defined according to the National Institute of Aging—Alzheimer’s Association criteria as not fulfilling the criteria for MCI or dementia^12^. Cognitively unimpaired participants consisted of cognitively healthy controls (*n* = 853) and participants with subjective cognitive decline (*n* = 329) who performed within normal ranges on a large cognitive test battery (that is, did not have MCI). The detailed study criteria have been published previously^46,52,53^. Briefly, the participants were aged 40–100 years, performed ≥24 points on the mini-mental state examination (MMSE) and spoke and understood Swedish to the extent that an interpreter was not necessary. None of the included participants fulfilled the clinical criteria for PD^54^ or prodromal DLB^42^ at baseline. All patients were enrolled and underwent baseline examination either from 2007 to 2015 (BioFINDER-1) or from 2017 to 2021 (BioFINDER-2). The exclusion criterion of MCI was defined as performing worse than −1.5 s.d. in at least one of the cognitive domains memory, attention/executive, verbal or visuospatial function. In BioFINDER-1 this was assessed by a senior neuropsychologist after a thorough neuropsychological battery, as described in detail previously^55^. In BioFINDER-2, MCI classification was operationalized as performing worse than −1.5 *z*-scores in any cognitive domain according to a regression-based norm accounting for age and education in Aβ-negative controls^56^ (see refs. ^57,58^ for a description on regression-based *z*-scores). Cognitive domain *z*-scores were derived by calculating the mean *z*-score of the tests in each of the following domains: attention/executive function (trail-making test A, trail-making test B and symbol digit modalities test (SDMT)), verbal ability (verbal fluency animals and the 15-word short version of the Boston naming test), memory (ten-word delayed recall from the Alzheimer’s disease assessment scale (ADAS)) and visuospatial (incomplete letters and cube analysis from the visual object and space perception battery) functions. Participants with subjective cognitive decline had subtle cognitive symptoms (perceived by participant or informant) but did not fulfill the criteria of MCI.

All participants provided written informed consent. Ethical approval was given by the Regional Ethical Committee in Lund, Sweden.

### Clinical outcomes

All clinical outcomes were *z*-scored according to the distribution of Aβ^–^ cognitively unimpaired participants in BioFINDER-1 and -2 except for the smell test, which was normalized using LB^–^ cognitively unimpaired participants in BioFINDER-1 and -2. The modified preclinical Alzheimer cognitive composite-5 (mPACC5, also referred to as PACC) was used as a measure of global cognition, containing tests of memory, executive, attention and verbal function^59^. It was calculated based on the previously described PACC5 using MMSE, SDMT and animal fluency^59^. Because the memory tests logical memory and the free and cued selective reminding tests were not available in BioFINDER, the ten-word delayed recall task from ADAS–cognition (ADAScog)^60^ was used (weighted twice), as previously applied in several studies^61,62^. The mPACC5 was thus calculated using *z*-scores based on the distribution in Aβ^–^ cognitively unimpaired in the following way: (MMSE + (ADAScog delayed recall × 2) + SDMT + animal fluency)/5.

Memory was measured using the ten-word delayed recall task from ADAScog^60^. Attention/executive function was measured using SDMT^63^ and, if that was not available, the serial 7s task of MMSE was used (see Supplementary Tables 2 and 3 for missingness)^64^.

Smell function was assessed in BioFINDER-1 using the brief smell identification test (Sensonics International)^65^ and in BioFINDER-2 using the very similar ODOFIN Burghart sniffin sticks (MediSense)^66^. To account for differences in maximum score (16 and 12, respectively), results were *z*-scored based on the distribution of LB^–^ participants in each cohort, separately.

Motor function was measured using the total score from the motor section of the informant-based cognitive impairment questionnaire (CIMP-QUEST)^67^, which assesses for example bradykinesia, changed way of walking, poorer balance, clumsier hands, tremor, changed facial expressions and dysarthria. In a supplementary analysis (Extended Data Fig. 2), motor function was measured using UPDRS-III (ref. ^68^). UPDRS-III was performed and rated by three certified physiotherapists.

### Biomarker of Aβ

Aβ positivity was determined as having abnormal [18 F]-flutemetamol PET using a predefined cutoff of 0.53 standardized uptake value ratio (SUVR) measured in a neocortical composite region using pons as a reference region, as previously described^46^. Due to the study design, Aβ-PET was not included at baseline in BioFINDER-1 and, here, abnormality was defined using a predefined CSF Aβ42/Aβ40 ratio cutoff of <0.066, as previously described^53^. Aβ42 and Aβ40 were analyzed on a Cobas E 601 analyzer using the Roche NeuroToolKit.

Both CSF and PET cutoff have previously been established using mixture modeling statistics^69^, and the two modalities have shown a very high concordance^70^.

### Biomarker of tau

Tau positivity was defined as either abnormal CSF P-tau217 (BioFINDER-1) or abnormal tau-PET (BioFINDER-2). CSF P-tau217 was measured using the Meso Scale Discovery platform using an assay developed by Eli Lilly, and tau-PET was performed using RO948 labeled with radioactive fluorine [18 F] as previously described^46^. SUVR was measured in a temporal metaregion of interest (ROI) using the inferior cerebellar cortex as reference region^46^. Cutoffs were established at mean + 2 s.d. in Aβ-negative controls as previously described; the cutoff for CSF P-tau217 was >11.42 pg ml^–1^ and for tau-PET >1.32 SUVR (ref. ^46^).

### Preparation of recombinant α-synuclein (LB pathology)

Purification of recombinant wild-type α-syn was performed as previously reported^10^, with minor modifications. Briefly, transformed *Escherichia coli* BL21 (DE3) bacteria (New England Biolabs) from a glycerol stock were streaked on a selective plate containing kanamycin (Kan+, 50 µg ml^–1^, Sigma) and incubated at 37 °C overnight. A single colony was selected and inoculated into 5 ml of Luria broth (LB, Sigma) with kanamycin and allowed to grow for 4–5 h at 37 °C with continuous agitation at 250 rpm. This starter culture was then added to 1 l of LB containing kanamycin and the overnight express autoinduction system (Merk-Millipore, no. 71300-4) in a fully baffled flask. Cells were grown in a shaking incubator at 37 °C, 200 rpm overnight. The following day the culture was split into four 250 ml flasks and centrifuged at 3,200*g* for 10 min at 4 °C. The pellet was gently resuspended in 25 ml of osmotic shock buffer containing 40% sucrose (Sigma), 2 mM EDTA (Sigma) and 30 mM Tris (Bio-Rad) at pH 7.2 using a serological pipette, and incubated for 10 min at room temperature under mild agitation on a rotator mixer. The solution was then centrifuged at 9,000*g*, 20 min at 20 s and 20 µl of saturated MgCl_2_ (Sigma) added. After 3 min incubation under mild rocking on ice the suspension was centrifuged at 9,000*g* for 30 min at 4 °C and the supernatant collected into a 100 ml glass beaker. pH was reduced to 3.5 by the addition of 400–600 µl HCl 1 M (PanReac AppliChem) and incubated under stirring for 10 min at room temperature. After a second centrifugation at 9,000*g* for 30 min at 4 °C, the supernatant was collected into a clean 100 ml glass beaker. pH was adjusted to 7.5 by the addition of 400–600 µl of NaOH 1 M (Sigma). The protein extract was filtered through a 0.22 µm filter (Merk-Millipore), loaded into a Ni–NTA column (Cytiva, no. 17525501) on an NGC chromatography system (Bio-Rad) and washed with 20 mM Tris pH 7.5 at room temperature. The column was further washed with 50 mM imidazole (Sigma) in Tris 20 mM pH 7.5, generating a peak that was not collected. A linear gradient up to 500 mM imidazole in 20 mM Tris pH 7.5 was performed, and the peak collected between 30 and 75% of imidazole buffer (150 and 375 mM, respectively). This peak was loaded onto a Q-HP anion exchange column (Cytiva, no. 17115401) and washed in Tris 20 mM pH 7.5, followed by another washing in 100 mM NaCl in Tris 20 mM pH 7.5. Again, a linear gradient up to 500 mM of NaCl in Tris 20 mM pH 7.5 was carried out to collect the peak between 300 and 350 mM NaCl. The fractions were pooled, filtered through a 0.22 µm filter and dialyzed against Milli-Q water overnight at 4 °C using a 3.5 kDa MWCO dialysis membrane (Thermo-Scientific). The following day, the protein was moved into fresh Milli-Q water and dialyzed for a further 4 h. Protein concentration was measured by spectrophotometry using a theoretical extinction coefficient at 280 nm of 0.36 (mg ml^–1^)–1 cm^–1^. Finally, the protein was lyophilized for 6 h and stored in aliquots at a final concentration of 1 mg ml^–1^ after resuspension into 500 µl of phosphate buffer (PB, 40 mM, pH 8.0, Sigma). Lyophilized aliquots were stored at −80 °C until usage.

### α-Syn RT-QuIC analyses

α-Syn RT-QuIC analyses were performed blinded to clinical status and diagnosis of the participant and according to an established protocol^9,10,71^, with minor modifications. Briefly, six 0.8 mm silica beads (OPS Diagnostics) per well were preloaded into black, clear-bottom, 96-well plates (Nalgene Nunc International). CSF samples were thawed and vortexed 10 s before use. Fifteen microliters of CSF was added to 85 μl of a reaction mix composed of 40 mM PB pH 8.0, 170 mM NaCl, 10 mM thioflavin-T (Sigma), 0.0015% SDS (Bio-Rad) and 0.1 g l^–1^ filtered recombinant α-syn (100 kDa Amicon centrifugal filters, Merck Millipore). Plates were closed with a plate sealer film (Nalgene Nunc International) and incubated into a Fluostar Omega plate reader (BMG Labtech) at 42 °C with intermittent double-orbital shaking at 400 rpm for 1 min, followed by 1 min rest. Fluorescence was measured every 45 min with 450 nm excitation and 480 nm emission filters during the 30 h test run. Samples and controls were run in quadruplicate and considered positive after the first run when at least three out of four replicates reached a threshold arbitrarily set at 30% of the median of Imax values reached by positive control replicates. To keep the risk of false positive results to a minimum, we repeated three times the analysis of samples showing seeding activity in only one or two out of four replicates in the first run. We considered a positive result only when at least four of the 12 total replicates reached threshold. We used 30 different batches of α-syn recombinant protein throughout the study, each undergoing a quality control test before use. We ran at least one positive and one negative control on each plate. Positive controls were chosen from patients with probable or definite DLB or PD whose CSF samples yielded four out of four positive replicates during screening. In each validated experiment (plate) included in the final analysis, the positive control/s showed at least three out of four positive replicates.

### Magnetic resonance imaging

Participants were examined using a Siemens 3 T Trio scanner (Siemens Medical Solutions) in BioFINDER-1 and a Siemens 3 T MAGNETOM Prisma scanner (Siemens Medical Solutions) in BioFINDER-2, as previously described^21,46^. T1 images underwent volumetric segmentation and parcellation using FreeSurfer (v.6.0, https://surfer.nmr.mgh.harvard.edu). The following regions were used in group comparisons (AD/LB) and independent effect of Aβ, tau and LB pathology: amygdala (left and right volumes); hippocampus (left and right volumes); medial temporal (left and right entorhinal, fusiform and parahippocampal cortical thicknesses); lateral temporal (left and right bank of superior temporal sulcus, inferior temporal, middle temporal, superior temporal, temporal pole and transverse temporal cortical thicknesses); medial parietal (paracentral, isthmus cingulate, posterior cingulate and precuneus cortical thicknesses); lateral parietal (postcentral, inferior parietal, superior parietal and supramarginal cortical thicknesses); occipital (cuneus, lateral occipital, lingual and pericalcarine cortical thicknesses); and frontal (remaining cortical FreeSurfer regions) areas. All but amygdala and hippocampus were surface weighted when constructing the composite region. Analyses were adjusted for age, sex and magnetic resonance camera (and also, in the case of hippocampus and amygdala volumes, total intracranial volume).

Moreover, all T1-weighted images were preprocessed using voxel-based morphometry running under the Statistical Parametric Mapping software (SPM12; https://www.fil.ion.ucl.ac.uk/spm/). First, images were segmented into gray matter, white matter and CSF. We then used the diffeomorphic nonlinear image registration tool^46^ to create a study-specific template based on the gray and white matter tissues of the whole sample. Once the template was created, gray matter tissues were warped into Montreal Neurological Institute space using individual flow fields resulting from registration, and voxel values were modulated for volumetric changes introduced by normalization. Finally the images were smoothed with an isotropic Gaussian kernel with 12 mm full-width at half-maximum. To account for differences in head size in statistical analyses, we calculated the total intracranial volumes of each subject as the sum of the gray matter, white matter and CSF volumes. In addition to analyses using the FreeSurfer-based ROIs described above, we also performed voxel-wise comparisons using analysis of variance on the smoothed gray matter images with group as a factor at four levels (AD^–^LB^–^, AD^–^LB^+^, AD^+^/LB^–^, AD^+^LB^+^) while controlling for age, sex, intracranial volume and magnetic resonance camera. All results were adjusted for multiple comparisons using a family-wise error rate correction set at *P* < 0.05.

### Statistical analyses

In cross-sectional analyses, AD/LB group, age, sex (assigned, not self-reported) and, for cognitive test outcomes, years of education, were used as independent variables in general linear regression models. When UPDRS-III was used as outcome the models were also adjusted for UPDRS-III rater (three raters in total). Dependent variables were either cognitive, smell or motor function. Next, binarized Aβ, tau and LB pathologies (to facilitate easier comparison of estimates) were used instead of AD/LB group. All three pathologies were used in the same model together with age, sex and years of education (education was not included in models of motor function). In longitudinal analyses, LME models were used (R packages lme4 and lmerTest). Cognitive function was used as outcome, and significant results are presented for the interactions AD/LB group × time and pathology × time. Models also included age, sex, years of education and random slopes and intercepts. For models including pathology × time, the interaction between time and all covariates was also included. In a sensitivity analysis, the LME models using AD/LB group × time were also adjusted for baseline cognitive test result and had the change from baseline in cognitive test result as outcome (for comparisons where there was a baseline group difference in cognition). All available data were used in statistical analyses. Missing data and number of participants at each visit are described in Supplementary Tables 2 and 3. A two-sided *P* < 0.05 was considered to indicate statistical significance. Multiple comparison corrections were performed using the false discovery rate method at *α* = 0.05, applying correction per outcome (that is, six comparisons for AD/LB group comparisons and three for the independent effects of LB, Aβ and tau pathology). Power calculations for linear regression models were performed using the R package WebPower (80% power, *α* = 0.05). Statistical analyses were performed using R v.4.1.

### Reporting summary

Further information on research design is available in the Nature Portfolio Reporting Summary linked to this article.

## Online content

Any methods, additional references, Nature Portfolio reporting summaries, source data, extended data, supplementary information, acknowledgements, peer review information; details of author contributions and competing interests; and statements of data and code availability are available at 10.1038/s41591-023-02450-0.

### Supplementary information

Supplementary Information Supplementary Figs. 1–3 and Tables 1–3.

Reporting Summary

## Figures and Tables

**Extended Data Table 1:**
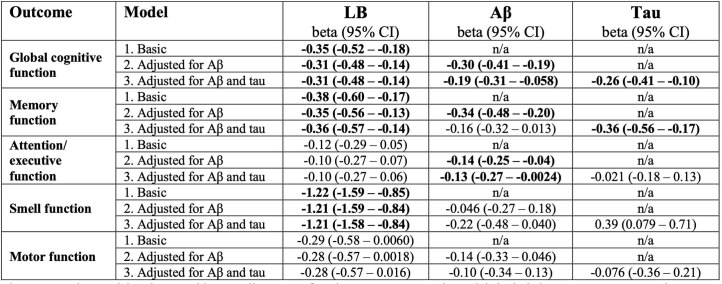
Cross-sectional association between pathology and baseline clinical outcome

Linear regression models using cognition, smell or motor function as outcome. Basic models included LB status, age, sex and years of education (education was not included in models with motor function) as predictors. At the second step, Aβ was added to the model and at the third step tau. The third model is thus the same as shown in Fig. 2F-J. Estimates in bold indicate a significant worse effect on the outcome. n/a, not applicable (not included in model)

**Extended Data Table 2:**
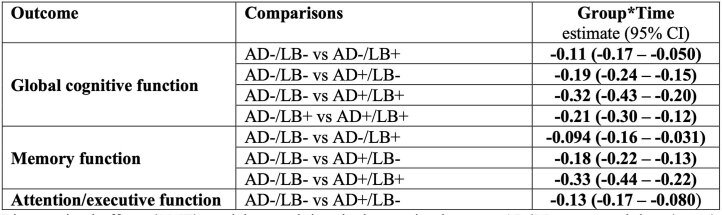
AD/LB group comparisons of change in cognition adjusted for baseline cognition

Linear mixed-effects (LME) models examining the interaction between AD/LB group and time (years) using change in cognition (global, memory or attention/executive) since baseline as outcome. The models were adjusted for baseline cognition (global, memory or attention/executive z-score), age, sex, and years of education, and included a random slope and intercept. The sensitivity analyses were performed in all instances where a baseline group difference was found (Fig. 2a–c).

**Extended Data Table 3:**
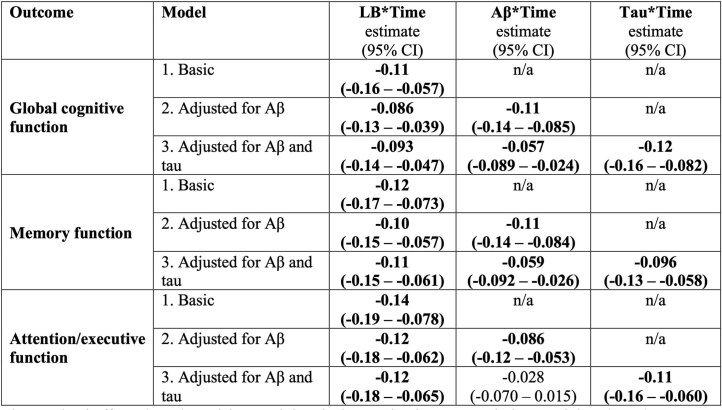
Longitudinal association between pathology and clinical outcome

Linear mixed-effects (LME) models examining the interaction between pathology and time (years) using cognition as outcome. Basic models included the interaction between time and LB pathology, age, sex and years of education, as well as a random slope and intercept. At the second step, Aβ was added to the model and at the third step tau. The third model is thus the same as shown in Fig. 3d–f.

**Extended Data Table 4:**
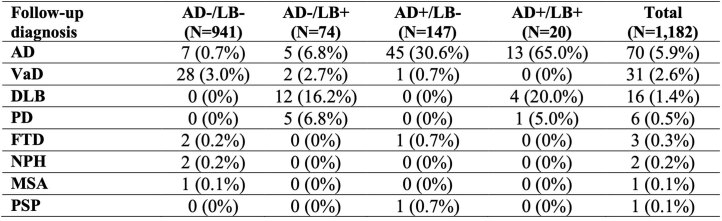
Progression to a clinical diagnosis during follow-up

Mean follow-up time: 4.46 (SD 2.68) years. For AD, VaD, DLB and FTD, event to clinical progression was determined as progression to at least MCI (that is, not requiring progression to dementia) together with the other clinical criteria. PD was diagnosed according to Gelb et al., but all also fulfilled the MDS criteria for probable PD at conversion. Abbreviations: AD, Alzheimer’s disease; DLB, dementia with Lewy bodies; FTD, frontotemporal dementia; MDS, Movement Disorder Society; MSA, multiple system atrophy; NPH, normal pressure hydrocephalus; PD, Parkinson’s disease; PSP, progressive supranuclear palsy; VaD, vascular disease/dementia

## Data Availability

Anonymized data will be shared by request from a qualified academic investigator for the sole purpose of replicating procedures and results presented in the article and providing data transfer is in agreement with EU legislation on the general data protection regulation and decisions by the Ethical Review Board of Sweden and Region Skåne, which should be regulated in a material transfer agreement.

## Code availability

No custom code or mathematical algorithm that was central to conclusions was used in this study.
