# Supplementary material for: Cognitive effects of Lewy body pathology in clinically unimpaired individuals
Source: Nat Med. 2023 Jul 18;29(8):1971–8. doi: 10.1038/s41591-023-02450-0 (PMC10427420; doi:10.1038/s41591-023-02450-0)
Supplement: Supplementary file 1 — Supplementary Figs. 1–3 and Tables 1–3. [file 41591_2023_2450_MOESM1_ESM.pdf]

nature medicine

Article

https://doi.org/10.1038/s41591-023-02450-0

Cognitive effects of Lewy body pathology in clinically unimpaired individuals

In the format provided by the authors and unedited

ONLINE SUPPLEMENT

SUPPLEMENTARY FIGURES

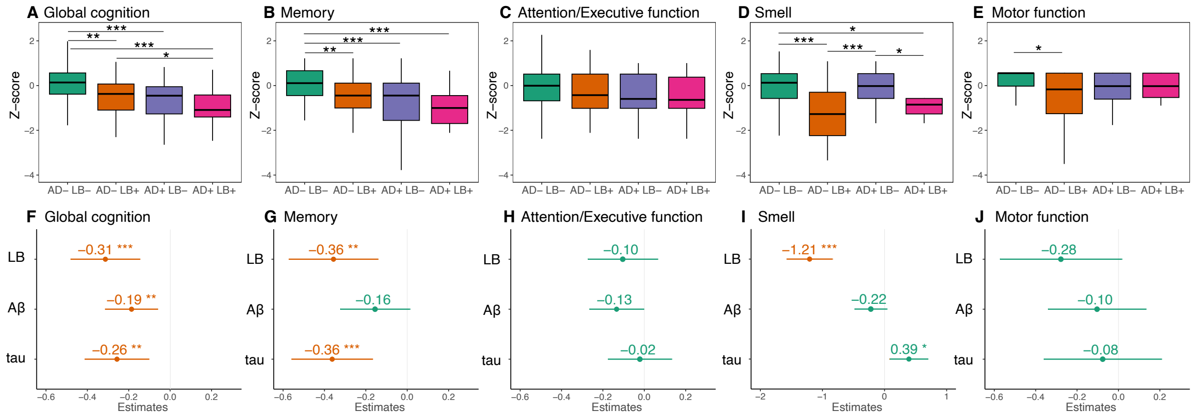

Supplementary Figure 1A-J. Comparisons between AD/LB groups and independent effects of LB, Aβ and tau pathologies on cross-sectional clinical outcomes with corrections for multiple comparisons. Significant effects (two-sided) were examined with linear regression models using two AD/LB groups (A-E) or all three pathologies binarized (F-J) in the same model (to examine independent effects), while adjusting for age, sex, and education (motor function was not adjusted for education). Outcomes were z-scored cognitive tests (A-C, F-H), smell identification test (D, I) and an informant-based motor questionnaire (E, J). Boxes in **A-E** show interquartile range, the horizontal lines are medians and the whiskers were plotted using the Tukey method. In **F-J,** the dot/center shows the estimate of the pathology and the error bars the 95% confidence interval. Red color in F-J indicated significant association between pathology and worse performance. Multiple comparison corrections were applied per outcome using the FDR method (at α=0.05). 941 participants were AD-/LB-, 74 AD-/LB+, 147 AD+/LB-, and 20 AD+/LB+. 94 were LB+, 304 Aβ+, and 195 tau+.

* p<0.05; ** p<0.01; *** p<0.001 (two-sided)

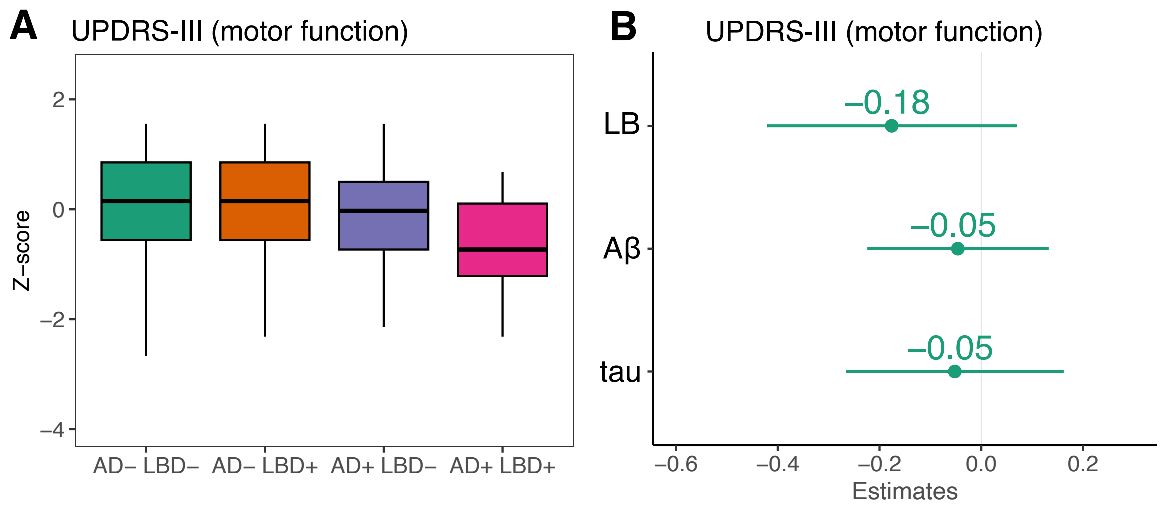

Supplementary Figure 2A-B. Comparison between AD/LB groups and independent effects of LB, Aβ and tau on motor function (UPDRS-III) with corrections for multiple comparisons. Significant effects (two-sided) were examined with linear regression models using two AD/LB groups (A) or all three pathologies binarized (B) in the same model (to examine independent effects), adjusted for age, sex, and UPDRS rater (three raters in total). The outcome was z-scored UPDRS-III values. Boxes in **A** show interquartile range, the horizontal lines are medians and the whiskers were plotted using the Tukey method. In **B,** the dot/center shows the estimate of the pathology and the error bars the 95% confidence interval. Multiple comparison corrections were applied per outcome using the FDR method (at α=0.05). No statistical analysis was significant. 660 participants had UPDRS data, of whom 510 AD-/LB-, 48 AD-/LB+, 90 AD+/LB-, and 12 AD+/LB+. 60 were LB+, 209 Aβ+, and 122 tau+.

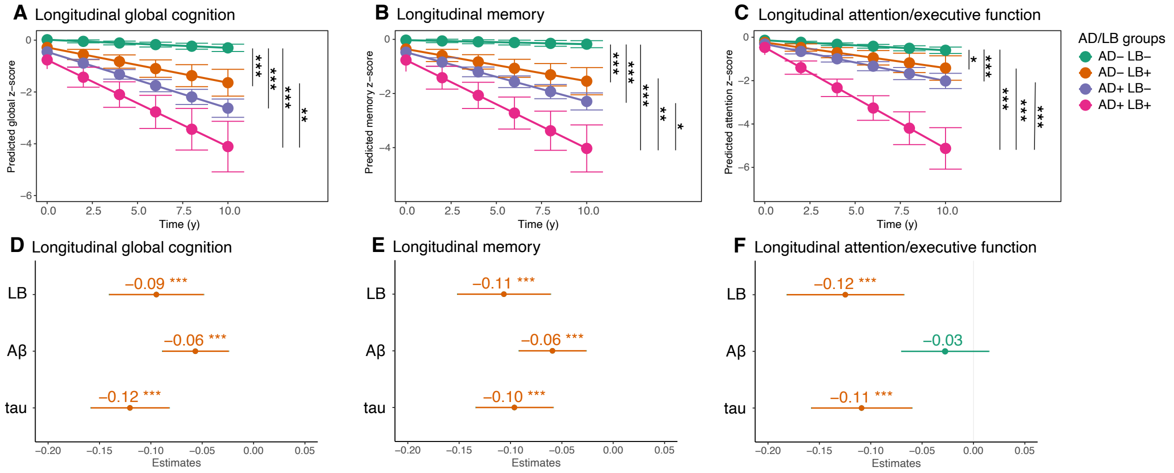

Supplementary Figure 3A-F. The independent effect of AD/LB groups and LB, Aβ and tau pathologies on longitudinal cognitive performance with corrections for multiple comparisons. Significant effects (two-sided) were examined with linear mixed-effects (LME) models focusing on the interaction AD/LB group*time, adjusted for age, sex, and education (A-C). The interaction time*all three pathologies (binarized) were used in the same model to examine the independent effects of each pathology on cognitive progression, while adjusting for age, sex, and education (D-F). Outcomes were z-scored cognitive tests. Red color in D-F indicated significant association between pathology and worse cognitive decline. Plots (**A-C**) show estimated marginal means and 95% confidence interval of the means obtained from linear mixed-effects models by AD/LB group. In **D-F**, the dot/center shows the interaction estimate of time*pathology and the error bars the 95% confidence interval. Multiple comparison corrections were applied per outcome using the FDR method (at α=0.05). 941 participants were AD-/LB-, 74 AD-/LB+, 147 AD+/LB-, and 20 AD+/LB+. 94 were LB+, 304 Aβ+, and 195 tau+.

* $p<0.05$; ** $p<0.01$; *** $p<0.001$ (two-sided)

SUPPLEMENTARY TABLES

Supplementary Table 1. Cross-sectional effects of LB, Aβ and tau pathologies on memory recognition

**Cognitive test**	**Pathology**	**Beta (95% CI)**
**RAVLT recognition**	LB	-0.75 (-1.81 – 0.30)
	Aβ	1.39 (0.13 – 2.64)
	Tau	**-1.97 (-3.26 – -0.68)**

Linear regression model including the predictors LB, Aβ, tau, age, sex, and education and the outcome Rey Auditory Verbal Learning Test (RAVLT) recognition, which measures the ability to recognize words from a previously presented word list of 16 words. Bold text indicates that the pathology is significantly associated with worse performance. RAVLT recognition was only available in a subsample of BioFINDER-1 participants (n=192).

Supplementary Table 2. Missing data in cross-sectional analyses (n=1,182)

Variables	% Missingness
Global cognitive function[1]	28.2%
Memory function[2]	0.3%
Attention/executive function[3]	0.3%
Symbol Digit Modalities Test	27.8%
MMSE Serial 7s	0.3%
Smell function[4]	66.3%
Motor function (CIMP-QUEST)[4]	52.7%[4]
Motor function (UPDRS-III)[4]	44.2%[4]

1. Measured using the mPACC5
2. Measured using the 10-word delayed recall test from ADAS-cog
3. Measured using the Symbol Digit Modalities Test if present, otherwise using the serial 7s subscore from the MMSE
4. Per study design, only available in a subset of participants

Supplementary Table 3. Missing data in longitudinal analyses (n=1,182)

Variables	Overall (3,839 data points)	Baseline (n=1,182)	1-year (n=279)[3]	2-year (n=856)	3-year (n=236)[3]	4-year (n=552)	5-year (n=40)[3]	6-year (n=255)	7-year (n=0)[3]	8-year (n=369)	9-year (n=0)[3]	10-year (n=70)
Global cognitive function	27.8%	28.2%	67.0%	11.5%	58.7%	13.3%	57.5%	2.4%	n/a	54.5%	n/a	8.6%
Memory function[1]	1.1%	0.3%	1.4%	0.9%	3.0%	2.0%	5.0%	0.4%	n/a	0.5%	n/a	2.9%
Attention/executive function[2]	0.3%	0.3%	0%	0%	0%	0.4%	5.0%	0.4%	n/a	0.5%	n/a	0%
Symbol Digit Modalities Test	17.3%	27.8%	31.5%	9.9%	30.6%	11.1%	22.5%	2.0%	n/a	2.2%	n/a	8.6%
MMSE Serial 7s	5.5%	0.3%	0.7%	0.2%	0.4%	0.5%	7.5%	0.4%	n/a	52.9%	n/a	0%

1. Measured using the 10-word delayed recall test from ADAS-cog
2. Measured using the Symbol Digit Modalities Test if present, otherwise using the serial 7s subscore from the MMSE.
3. Per study design, controls were followed every other year (hence the low number of participants at odd years), while SCD participants were followed every year.

n, number of participants
