# Supplementary material for: Cognitive effects of Lewy body pathology in clinically unimpaired individuals
Source: Nat Med. 2023 Jul 18;29(8):1971–8. doi: 10.1038/s41591-023-02450-0 (PMC10427420; doi:10.1038/s41591-023-02450-0)
Supplement: Supplementary file 2 — Reporting Summary [file 41591_2023_2450_MOESM2_ESM.pdf]

Corresponding author(s): Piero Parchi
Oskar Hansson

Last updated by author(s): May 26, 2023

Reporting Summary

Nature Portfolio wishes to improve the reproducibility of the work that we publish. This form provides structure for consistency and transparency in reporting. For further information on Nature Portfolio policies, see our Editorial Policies and the Editorial Policy Checklist.

Statistics

For all statistical analyses, confirm that the following items are present in the figure legend, table legend, main text, or Methods section.

n/a	Confirmed	
☐	☒	The exact sample size (*n*) for each experimental group/condition, given as a discrete number and unit of measurement
☐	☒	A statement on whether measurements were taken from distinct samples or whether the same sample was measured repeatedly
☐	☒	The statistical test(s) used AND whether they are one- or two-sided<br>*Only common tests should be described solely by name; describe more complex techniques in the Methods section.*
☐	☒	A description of all covariates tested
☐	☒	A description of any assumptions or corrections, such as tests of normality and adjustment for multiple comparisons
☐	☒	A full description of the statistical parameters including central tendency (e.g. means) or other basic estimates (e.g. regression coefficient) AND variation (e.g. standard deviation) or associated estimates of uncertainty (e.g. confidence intervals)
☐	☒	For null hypothesis testing, the test statistic (e.g. F, t, r) with confidence intervals, effect sizes, degrees of freedom and P value noted<br>*Give P values as exact values whenever suitable.*
☒	☐	For Bayesian analysis, information on the choice of priors and Markov chain Monte Carlo settings
☒	☐	For hierarchical and complex designs, identification of the appropriate level for tests and full reporting of outcomes
☐	☒	Estimates of effect sizes (e.g. Cohen's *d*, Pearson's *r*), indicating how they were calculated

Our web collection on statistics for biologists contains articles on many of the points above.

Software and code

Policy information about availability of computer code

Data collection	No software was used.
Data analysis	R version 4.1 using packages lme4 (ver 1.1), lmerTest (ver 3.1), and WebPower (ver 0.8).<br>FreeSurfer image analysis pipeline v6.0 (see http://surfer.nmr.mgh.harvard.edu/)

For manuscripts utilizing custom algorithms or software that are central to the research but not yet described in published literature, software must be made available to editors and reviewers. We strongly encourage code deposition in a community repository (e.g. GitHub). See the Nature Portfolio guidelines for submitting code & software for further information.

Data

Policy information about availability of data

All manuscripts must include a data availability statement. This statement should provide the following information, where applicable:

- Accession codes, unique identifiers, or web links for publicly available datasets
- A description of any restrictions on data availability
- For clinical datasets or third party data, please ensure that the statement adheres to our policy

Anonymized data will be shared by request from a qualified academic investigator for the sole purpose of replicating procedures and results presented in the article and as long as data transfer is in agreement with EU legislation on the general data protection regulation and decisions by the Ethical Review Board of Sweden and Region Skåne, which should be regulated in a material transfer agreement.

Human research participants

Policy information about studies involving human research participants and Sex and Gender in Research.

Reporting on sex and gender	The study includes both men and women. Sex was assigned (not self reported). 698 (59.1%) were female. Sex was adjusted for in the statistical models.
Population characteristics	Detailed information is given in Table 1, Extended Data Table 1 and Online Methods. In short, we present results for analyses from the BioFINDER-1 and BioFINDER-2 cohorts, both with very similar demographics. Cognitively unimpaired participants were included in the present study (n=1,182) with cross-sectional and longitudinal data. The cognitively unimpaired participants consisted of cognitively healthy controls (n=853) and participants with subjective cognitive decline (SCD; n=329) who performed within normal ranges on a large cognitive test battery (i.e., did not have MCI). The mean (SD) age was 70 (8.8) years and 69% (n=698) were women.
Recruitment	This project was done as part of the prospective Swedish BioFINDER study. All patients were recruited from the Southern part of Sweden and underwent baseline examination from 2007 to 2015 (BioFINDER-1) or from 2017 to 2021 (BioFINDER-2). Controls in BioFINDER-1, were consecutively recruited from the population-based Malmö Diet and Cancer Study. Controls in BioFINDER-2, were recruited through advertisment. Cognitively unimpaired participants with subjective cognitive decline (SCD) in both BioFINDER-1 and BioFINDER-2 were recruited from the memory clinics of Skåne University Hospital and Ängelholm hospital, Sweden. The recruitment is described in detail in reference 47 and Methods-only reference 5. Per study design, only those that speak Swedish were included, which might reduce the number of foreign-born participants.
Ethics oversight	The study was approved by the Regional Ethics Committee in Lund, Sweden. All participants gave their informed consent to participate in the study and the data were collected according to the Declaration of Helsinki.

Note that full information on the approval of the study protocol must also be provided in the manuscript.

Field-specific reporting

Please select the one below that is the best fit for your research. If you are not sure, read the appropriate sections before making your selection.

☒ Life sciences ☐ Behavioural & social sciences ☐ Ecological, evolutionary & environmental sciences

For a reference copy of the document with all sections, see nature.com/documents/nr-reporting-summary-flat.pdf

Life sciences study design

All studies must disclose on these points even when the disclosure is negative.

Sample size	The study included a large sample size (n=1,182 participants with cross-sectional data and 3,839 data points for the longitudinal analysis). For statistical analyses using outcomes with substantial missing data, power analyses were performed. For example, there were n=660 with UPDRS (motor function) data and n=854 with Symbol Digit Modalities Test data. Power calculation at 80% power and α=0.05 showed that we were powered to detect small or very small effect sizes (f2=0.012 and f2=0.0092, respectively).
Data exclusions	Analyses were performed on all eligible participants and not on a restricted sample with complete data for all cognitive and non-cognitive measures. The rationale for this was to not introduce a selection bias.
Replication	Two independent cohorts were used in the study. Data was pooled to achieve better statistical power and no replication in a third cohort was perform.
Randomization	In these 2 cohort studies (observational studies) no allocation into experimental groups were performed, therefore randomization is not relevant to this study. Statistical analyses were controlled for potential confounding effects of age, sex and education.
Blinding	Diagnostic assessments and all test measures were performed blinded to the α-synuclein SAA results.

Reporting for specific materials, systems and methods

We require information from authors about some types of materials, experimental systems and methods used in many studies. Here, indicate whether each material, system or method listed is relevant to your study. If you are not sure if a list item applies to your research, read the appropriate section before selecting a response.

Materials & experimental systems

n/a	Involved in the study
☐	☒ Antibodies
☒	☐ Eukaryotic cell lines
☒	☐ Palaeontology and archaeology
☒	☐ Animals and other organisms
☐	☒ Clinical data
☒	☐ Dual use research of concern

Methods

n/a	Involved in the study
☒	☐ ChIP-seq
☒	☐ Flow cytometry
☒	☐ MRI-based neuroimaging

Antibodies

Antibodies used

Phosphorylated tau 217(p-tau217) assay.

Validation

The phosphorylated tau 217 (p-tau217) assay in CSF was performed using phospho-specific biotinylated capture antibody (IBA493, developed by Lilly Research Laboratories) and SULFO-TAG- conjugated anti-tau detection antibody (4G10E2, developed by Lilly Research Laboratories). The p-tau217 immunoassay has been fully described by Palmqvist et al. (JAMA. 2020;324(8):772-781).

Clinical data

Policy information about clinical studies

All manuscripts should comply with the ICMJE guidelines for publication of clinical research and a completed CONSORT checklist must be included with all submissions.

Clinical trial registration

NCT01208675 and NCT03174938

Study protocol

BioFINDER-1: https://clinicaltrials.gov/ct2/show/NCT01208675
BioFINDER-2: https://clinicaltrials.gov/ct2/show/NCT03174938

Data collection

All patients were recruited from the Southern part of Sweden and underwent baseline examination from 2007 to 2015 (BioFINDER-1) or from 2017 to 2021 (BioFINDER-2). Biomarker data was collected at baseline. Data were collected at the Memory Clinic of Skåne university Hospital, Sweden, and the Memory Clinic of Ängelholm Hospital, Sweden.

Outcomes

Primary outcomes were cognitive test scores (global cognition, attention/executive function, memory), smell function (measured using a smell test), and motor function (using the CIMP-QUEST questionnaire). Secondary outcome was motor function using the UPDRS-III scale.
